# Human Milk Bioactive Compounds and Psychomotor Outcomes in Infants and Children: A Systematic Review

**DOI:** 10.1002/mnfr.70553

**Published:** 2026-07-23

**Authors:** Lara Huber, Eduard Flores Ventura, Regina Ensenauer, Daria Guseva

**Affiliations:** ^1^ Department of Child Nutrition Max Rubner‐Institut – Federal Research Institute of Nutrition and Food Karlsruhe Germany; ^2^ Department of Biotechnology Institute of Agrochemistry and Food Technology – Spanish National Research Council (IATA‐CSIC) Valencia Spain; ^3^ Executive body National Breastfeeding Committee Max Rubner‐Institut – Federal Research Institute of Nutrition and Food Karlsruhe Germany

**Keywords:** bioactive compounds, breastfeeding, human milk, carotenoids, HMOs, polar lipids, proteins, psychomotor development, PUFAs, SFAs

## Abstract

Human milk (HM) provides nutrients and bioactive compounds (BCs) essential for healthy child development. This systematic review aimed to evaluate the short‐ and long‐term effects of individual groups of BCs on psychomotor outcomes, including motor, cognitive, language, and executive functions. A comprehensive literature search was conducted in MEDLINE (PubMed), Scopus, Web of Science, and Embase in accordance with Preferred Reporting Items for Systematic Reviews and Meta‐Analyses (PRISMA) guidelines. Eligibility was defined using the PECOTS framework, focusing on healthy, term, breastfed children with follow‐up up to 18 years. Study quality was assessed with the Joanna Briggs Institute (JBI) tool and Cochrane Risk of Bias (RoB 2) tools. Eighteen studies met the inclusion criteria. The most consistent evidence was for polyunsaturated fatty acids (PUFAs), particularly n‐3 PUFAs, which were generally associated with favorable psychomotor outcomes throughout infancy and later childhood. In contrast, higher levels of n‐6 PUFAs showed less consistent or potentially unfavorable associations. Among HM oligosaccharides, fucosylated HMOs were consistently associated with improved cognitive and language‐related outcomes, whereas evidence for other HMO groups was limited and heterogeneous. Findings related to proteins, phospholipids, sphingolipids, and carotenoids were inconsistent. HM BCs, particularly n‐3 PUFAs and fucosylated HMOs, may support psychomotor development in infancy, preschool age, and school age. However, the overall evidence remains heterogeneous. Further research using standardized methodologies is required to elucidate long‐term effects and underlying mechanisms.

## Graphical abstract

In our review, we assess the role of HM fatty acids, oligosaccharides, proteins, and carotenoids, on psychomotor development in children up to 18 years. Findings suggest that HM BCs, particularly n‐3 PUFAs and fucosylated HMOs, might contribute to a favorable psychomotor development in infancy, preschool age, and school age.

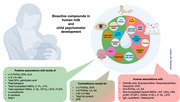

## 1 Introduction

Early childhood is a critical period for neurodevelopment, when nutritional exposures can profoundly shape long‐term functional outcomes [1, 2]. During this phase, human milk (HM) is the optimal source of nutrition for infants and has been consistently associated with benefits for physical health, psychomotor, and cognitive development [3, 4, 5]. Based on a large and growing body of evidence supporting these and further health benefits, the World Health Organization (WHO), the United Nations Children's Fund (UNICEF), and the German Association of the Scientific Medical Societies recommend starting breastfeeding within the first hour after birth and endorse exclusive breastfeeding for the first 6 months [6, 7].

Psychomotor development refers to the progressive maturation of a child's motor, cognitive, emotional, and social abilities from early life through adolescence [8]. These domains are interconnected and share common neurobiological pathways [9]. Previous research has primarily examined the effects of individual bioactive compounds (BCs) found in human milk, including polyunsaturated fatty acids (PUFAs) [10], HM oligosaccharides (HMOs) [11], as well as polar lipids, proteins, hormones, and carotenoids, on early development and psychomotor outcomes [12, 13, 14]. In particular, PUFAs have been suggested to relate to neurodevelopmental outcomes in early life. HMOs, which act as prebiotics, shape the infant gut microbiota [12, 15] and influence immune function [16], growth, and neurodevelopment [17, 18, 19, 20, 21]. Their role in the gut‐brain axis has been linked to 2’‐FL‐mediated production of short‐chain fatty acids (SCFAs), which may impact neurotransmitter balance [22]. However, a comprehensive analysis of the existing evidence on psychomotor development in children up to 18 years old, linked to the potential synergistic effects of BCs in human milk, is still lacking.

The purpose of our review was to summarize and analyze the results from human studies investigating the effects of individual groups of BCs on psychomotor development from infancy up to 18 years of age. Furthermore, we aimed to identify methodological strengths and limitations of the included studies in order to emphasize the need for standardization in future human milk research.

## 2 Methods

A comprehensive literature search was conducted in MEDLINE (PubMed), Scopus, Web of Science, and Embase, according to the Preferred Reporting Items for Systematic Reviews and Meta‐Analyses (PRISMA) guidelines [23]. These databases were selected to ensure comprehensive coverage of biomedical, clinical, and interdisciplinary literature relevant to breastfeeding and the role of individual HM BCs in the psychomotor development of children from birth to 18 years of age. The review, as part of a larger project was registered in PROSPERO under protocol CRD42023432030. Data synthesis was performed on original studies focused specifically on short‐ and long‐term effects of HM BCs on children's psychomotor development.

### 2.1 Inclusion and Exclusion Criteria Screening Process

The PECOTS strategy was used to develop generic search strings (Table S1). The eligibility criteria were defined to address the review question: how the levels of individual groups of BCs are associated with psychomotor outcomes in children from birth up to 18 years of age. The criteria included: (P) Population – healthy term children up to 18 years; (E) Exposure – HM BCs; (C) Comparison – internal comparison group of children; (O) Outcome – psychomotor child development; (T) Time – follow‐up < 18 years; (S) Study design – original observational studies, cohort studies, randomized controlled trials (RCTs); studies written in English. Studies were excluded if they: (1) included preterm infants, (2) investigated non‐human populations (animal studies), (3) investigated very low birth weight infants, (4) did not analyze HM BCs, (5) did not report psychomotor outcomes in children, (6) were narrative reviews, systematic reviews, commentaries, or editorials, or (7) were not available in English. Generic search strings were adapted for each database (Table S2). The search was performed on July 24, 2024. Identified articles were exported into the online platform Rayyan (https://rayyan.ai), duplicates were resolved, and two researchers independently screened them by title and abstract. Conflicts and disagreements were resolved by a third participant. The full texts were reviewed to identify articles that met the inclusion criteria. Additionally, the reference lists of all included studies and relevant reviews were hand‐searched to identify further eligible publications.

### 2.2 Data Extraction, Synthesis, and Analysis

Initial data extraction from the included studies was performed independently by two researchers using a developed data extraction table to record data as follows: reference/year, country, study design, characteristics of the studied population, methods for HM collection, HM BCs being studied, HM sampling time, analytical method, outcome being measured (characteristics to assess psychomotor development), time point for outcome measurement from birth, tool/intervention used for measuring the outcome, summary of the results/effects of the exposure, and confounding factors. The Excel‐based data extraction form was initially pilot‐tested on three studies and subsequently refined by two authors through the addition of missing variables, clarification of definitions, and removal of redundant fields. Any disagreements during pilot testing were resolved through discussion between the authors. The relationships between exposure and the investigated variables were classified as direct, inverse, or no association based on the results of statistical analyses and corresponding effect sizes (β or r coefficients) and included in Table 1 (Study Characteristics). This classification considered both the direction of the observed effects and their statistical significance, with a significance level set at *p* < 0.05. A lollipop diagram was created in R using the referevbnce table (Table S7) and completed in PowerPoint.

**TABLE 1:** Characteristics of the included studies.

Reference, year published	Country	Design	Number of mother‐child pairs	HM bioactive compounds	HM sampling time	Time point for outcome measurement from birth	Outcome	Effects & associations	Adjustment for
**PUFAs**									
Agostini et al., 2001 [30]	Italy	L‐P	Gender not specified (*n* = 44)	DHA, AA, total n‐6 LC‐PUFA, n‐3 LC‐PUFA	Birth, 1, 3, 6, 9, 12 mo	12 mo	Mental and psychomotor development	Positive association between HM fat content at 6 mo and MDI at 12 mo (*r* = 0.59, *p* = 0.001); ;No association between HM fat content and PDI scores at any time point	Maternal age, parity, education, social class, usual smokers
Bernard et al., 2015 [29]	France	L‐P	Gender not specified (*n* = 709)	LA, AA, total n‐6 PUFA, total n‐6 LC‐PUFA, ALA, EPA, DHA, total n‐3 PUFA, total n‐3 LC‐PUFA, LA/ALA ratio, AA/DHA ratio	3.9 d + 1.1 d	2, 3 y	Motor development, child language ability, cognition	No associations between AA or DHA levels in HM in colostrum and Motor‐2 at 2 y and ASQ‐3 at 3 y; Inverse association between LA levels in HM in colostrum and Motor‐2 at 2 y (β = 0.10, 95 % CI: ‐0.20; −0.01) and ASQ‐3 at 3 y (β = −1.9, 95 % CI: −3,1, −0.8)	Maternal age at conception, mother's prepregnancy BMI, smoking status, alcohol consumption during pregnancy, recruitment center, household income, parental education, gestational age, child sex, age at assessment, sibling, maternal activities with child, main daytime caregiver, pre‐elementary schooling
Dalmeijer et al., 2016 [31]	Netherlands	L‐P	Boys = 73 Girls = 84	DHA, EPA, AA, n‐3 LC‐PUFAs, DHA/AA ratio, EPA/AA ratio	3 mo	12 y	School performance	Positive association between total n‐3 LC‐PUFAs in HM at 3 mo and Cito‐test score for girls at 12 y (β = 2.96, 95 % CI: 0.24; 5.69); Positive association between DHA in HM at 3 mo and Cito‐test score and teacher school advice for girls at 12 y (β = 7.09, 95 % CI: 0.9; 13.3); No association between LC‐PUFA content in HM at 3 mo and school performance in boys at 12 y	Maternal age, maternal smoking during pregnancy, parental education level, gestational age in weeks, sex of the child, birth weight of the child, children's mental health at 12 y of age, fish consumption
Dunstan et al., 2007 [28]	Australia	RCT	Gender not specified (*n* = 98)	EPA, DHA, DPA, AA	3 d, 6 wk, 6 mo	2.5 y	Mental development, receptive language, behavior	Positive association between EPA (r = 0.405, *p* = 0.001) and DHA (*r* = 0.315, *p* = 0.016) in HM at 3 d and 6 mo and eye‐hand coordination, general quotient at 2.5 y; Inverse association between AA in HM at 6 mo and vocabulary skills as measured by the average length of phrase used (r = ‐0.533, *p* = m0.002) and the number of words used (*r* = −0.371, *p* = 0.028) at 2.5 y	Maternal age, pre‐pregnancy BMI, maternal allergy status, parity, gestational length, neonatal anthropometric measurements, feeding method at 1 y
Guxens et al., 2011 [34]	Spain	L‐P	Gender not specified (*n* = 504)	n‐3 PUFAs, ALA, EPA, DPA, DHA, n‐6 PUFAs, LA, GLA, DGLA, AA, ADA, OA, n‐3/n‐6 PUFAs	48 to 96 h	14 mo	Mental development	Positive association between total n‐3 PUFAs (β = 1.76, 95 % CI: ‐0.88 – 4.40) and n‐3/n‐6 PUFAs ratio (β = 2.00, 95 % CI: ‐0.78 – 4.78) in colostrum and infant mental score at 14 mo	Maternal age, parental education, parental social class, parental IQ, parental mental health, parental attachment to the child, maternal alcohol use during pregnancy, persistent toxic compounds, maternal use of gas cooking at home during pregnancy, children's exact age at testing, age of solid food introduction, psychologist administering the test, quality of neuropsychological testing
Hahn‐Holbrook et al., 2019 [39]	United States	L‐P	Gender not specified (*n* = 52)	n‐3 PUFAs, n‐6 PUFAs, n‐3/n‐6 PUFAs ratio	3 mo	3, 6 mo	Temperament	No association between n‐6/n‐3 PUFAs ratio, n‐6 PUFA in HM at 3 mo and temperament at 3 mo; Inverse association between n‐3 PUFA in HM at 3 mo and *negative affectivity dimension* at 3 mo (β = −0.352, *p* = 0.020)	Maternal age, maternal prepregnancy BMI, weight gain in pregnancy, parity, maternal race/ ethnicity, maternal education, marital status, household income, gestational age at birth, infant sex, exclusively breastfeeding status, infant BMIP at birth and at 3 mo
Hurtado et al., 2015 [33]	Spain	RCT	Gender not specified (n = 110)	DHA, EPA, SFAs, MUFAs, n‐6 PUFAs, n‐3 PUFAs, OA, LA, ALA, NA	Birth, 1, 2, 4 mo	2.5, 7.5, 12 mo	Visual and neurodevelopment	No association between n‐3 PUFAs in HM and visual development at 2.5 and 7.5 mo and neurodevelopment at 12 mo	Maternal diet, gestational age, sex of the infant, perinatal conditions
Siziba et al., 2018 [44]	South Africa	C‐S	Gender not specified (*n* = 353)	DHA, AA, DGLA, AdA, LA, ALA, EPA, DPA, n‐3 PUFAs, n‐6 PUFAs, n‐3/n‐6 PUFAs ratio, LC‐PUFAs	6 mo	6 mo	Locomotor skills, eye‐hand coordination	Positive association between n‐3 LC‐PUFAs in HM and locomotor (β = 0.356; *p* = 0.024) as well as eye‐hand coordination (β = 0.467; *p* = 0.014) at 6 mo; Inverse association between n‐6 LC‐PUFAs in HM and locomotor (β = 0.356; *p* = 0.024) as well as eye‐hand coordination (β = 0.467; *p* = 0.014) at 6 mo	Child sex, age, Hb, LAZ
Xiang et al., 2000 [35]	Sweden	L‐P	Gender not specified (*n* = 19)	AA, DHA, LA, LNA, n‐3 PUFAs, n‐6 PUFAs	Birth, 1, 3 mo	1, 3 mo	BRW, OFC	Positive association between AA/DHA ratio in HM during the first 3 mo and OFC, and BRW at 1 mo (*p* < 0.01) and 3 mo (*p* < 0.01); No association between LA or LNA in HM during the first 3 mo and OFC or BRW at 1 mo or 3 mo; No association between AA or DHA in HM during the first 3 mo and OFC or BRW at 1 mo or 3 mo	NA
Zielinska et al., 2019 [14]	Poland	L‐P	Gender not specified (*n* = 53)	n‐3 PUFAs, n‐3/n‐6 PUFAs ratio, LA, ALA, DHA, EPA, AA	1, 3, 6 mo	6 mo	Psychomotor development	Positive association between DHA (β = 0.275; p < 0.05), ALA (β = 0.432; p < 0.05), n‐3 PUFAs (β = 0.423; p < 0.05) in BM during the first 3 mo with motor skills at 6 mo; Positive association between DHA in HM during the first 3 mo and the *Perception subscale* at 6 mo (β = 0.316; p < 0.05)	Parental age, parental education level, marital status, maternal psychological status, maternal nutrition, household income, children in the household, gestational age, birth parameters, gender, child anthropometric parameters, child nutrition
**Polar lipids and SFAs**									
Li et al., 2023 [41]	]United States	C–C	Boys = 40 Girls = 42	Phospholipids, ceramides, amino acids, neurotransmitters, NAD+, PA, DeoxyCer, hexanolycarnitine, DHC, plasmalogens	4.6 mo ± 2.5 mo	14.2 mo ± 3.1 mo	Neurocognitive development	Positive association between levels of plasmalogen (p16:0/20:4) and NAD^+^ in HM at 4.6 ± 2.5 mo and ASQ2 scores at 14.2 + 3.1 mo for the male risk group of NDD; Inverse association between DeoxyCer (m18:1/24:1), hexanoylcarnitine, PA (16:1/16:1) in HM at 4.6 ± 2.5 mo and ASQ2 scores at 14.2 + 3.1 mo for female group of NDD (AUC: 0.81; 95 % CI: 0.66 ‐0.96; *p* = 0.003); Inverse association between Butyrylcarnitine, DHC (18:1/ 24:0) in HM at 4.6 ± 2.5 mo and ASQ2 score at 14.2 + 3.1 mo for the male risk group of NDD	Prematurity, low birth weight, perinatal difficulties, socio‐economic variables
Ramadurai et al., 2022 [42]	United States	L–P	Gender not specified (*n* = 39)	pPE(16:0p/18:1)‐H, pPE(16:0p/18:2)‐H, pPE(18:0p/18:1)‐H, pPE(18:0p/18:2)‐H, pPE(18:1p/18:2)‐H, pPE(16:0p/20:4)‐H, pPE(18:0p/20:1)‐H, pPE(18:0p/20:3)‐H, pPE(16:0p/22:4)‐H, pPE(18:0p/20:4)‐H, pPE(18:1p/20:4)‐H, pPE(16:0p/22:6)‐H, pPE(18:0p/22:4)‐H, pPE(18:0p/22:5)‐H, pPE(18:0p/22:6)‐H	1, 4 mo	1 to 9 mo	Neuro‐development	No association between pPE in HM and infant gross motor, fine motor, social developmental milestones (age of rolling from front to back, rolling from back to front, transferring objects between hands, smilling) at 1 and 4 mo	Maternal BMI, maternal race, maternal FAs intake, number of days postpartum
Siziba et al., 2019 [44]	South Africa	C‐S	Gender not specified (n = 353)	SFA, C16:1	6 mo	6 mo	Locomotor skills, eye‐hand coordination	Positive association between C16:1, SFA levels in HM and locomotor development at 6 mo (β = 0.316; *p* = 0.046);	Child sex, age, Hb, LAZ
**HMOs**									
Berger et al., 2020 [36]	United States	L‐P	Gender not specified (*n* = 50)	2’‐FL, 3‐FL, 3’‐SL, 6’‐SL, DFLac, LNT, LNnT, LNFP I, LNFPII, LNFPIII, LST b, LSTc, DFLNT, DSLNT, LNH, FLNH, DFLNH, FDSLNH, DSLNH	1, 6 mo	24 mo		Positive association between 2’‐FL at 1 mo in HM and infant cognitive development scores at 24 mo (β = 0.59; *p* < 0.01); No association between 2’‐FL in HM at 6 mo and infant cognitive development at 24 mo	Maternal age, pre‐pregnancy BMI, secretor status, education level, infant age, infant sex, infant birth weight
Cho et al., 2021 [37]	United States	L‐P	Gender not specified (*n* = 99)	2’‐FL, 3‐FL, A‐tetra, 3’‐SL, 6’‐SL, LNT, LNNT, LNFP‐I	2 to 25 mo	2 to 25 mo	Cognitive development	Positive association between 3’‐SL in HM at 2 to 25 mo and language abilities between 2 and 25 mo (*p* = 0.002; effect size (EF), 13.12; 95% CI: 5.36–20.80) in the A‐tetra+ group	Maternal age, maternal education, socioeconomic status, gestational age, infant sex, breastfeeding duration, A‐tetra+ and A‐tetra‐ HM
Cho et al., 2023 [38]	United States	L‐P	Gender not specified (*n* = 105)	2’‐FL, 3‐FL, 3’‐SL, 6’‐SL, LNT, LNnT, LNFP‐I, A‐tetra	8.09 mo + 2.48 mo	8.09 mo + 2.48 mo	Cognitive development	Positive association between 3‐FL in HM at 8.09 + 2.8 mo and gross motor (*p* = 0.027) and visual reception at 8.09 + 2.8 mo (*p* = 0.041); Positive associations with 3´‐SL and *Bif. Bifidum* (*p* = 0.01), 6´‐SL and *B. fragilis* (p = 0.019), and LNFP‐I and *Bif. Kashiwanohense* (*p* = 0.048) at 8.09 + 2.8 mo and expressive language at 8.09 + 2.8 mo; Inverse associations with LNT and *Bif. Breve* (*p* = 0.011) and LNT and *Bif. Longum* (*p* = 0.022) at 8.09 + 2.8 mo and expressive language at 8.09 + 2.8 mo	Infant sex, mode of delivery, maternal education level, site and batch of HMO analyses, A‐tetra+ and A‐tetra‐ HM
Ferreira etal, 2021 [43]	Brazil	L‐P	Gender not specified (*n* = 74)	3FL, 3′SL, 6′SL, DFLac, DFLNH, DFLNT, DSLNH, DSLNT, FDSLNH, FLNH, LNFP, LNFP‐I, LNFP‐II, LNFP‐III, LNH, LNnT, LNT, LSTb, LSTc, Sia, Fuc	1, 6, 12 mo	1, 6, 12 mo	Cognitive development	Inverse association between LNT in HM and infant's risk of inadequate development for personal‐skills (β = 0.06, 95 % CI: 0.01 ‐0.76), > developmental domains (β = 0.06, 95 % CI: 0.01 ‐ 0.59) at 1, 6, 12 mo	Maternal age, maternal prepregnancy BMI, gestational weight gain, parity, maternal education, gestational age at birth, mode of breastfeeding at 1 mo
Jorgensen et al., 2021 [40]	United States	L‐P	Gender not specified (*n* = 659)	3’‐SL, 6’‐SL, 3‐FL, 2’‐FL, LDFT, LNT, LNnT, LNT + LNnT, LSTa, LSTb, LSTc, LNFP‐I + LNFP‐III, LNFP‐II, F‐LSTc, LNDFH + 3120, 4100a, 4100b, LNH, LNnH, p‐LNH3, S‐LNH, 4021a + S‐LNnH‐II, MFpLNH‐IV, 4120a, MFLNH‐I + MFLNH‐III, IFLNH‐III, IFLNH‐I, 4211a, 4211b, 4211c, DFLNHa, DFLNHb, DFLNHc, DFpLNH‐II, DFS‐LNnH, TFLNH, 4240a, 4320a, 5130a, 5130b, 5130c, 5230a + DFLNnO, I/DFLNO‐II, 5230a, 5230b, 5300a4, F‐LNO, 5311a, DFLNO‐I, DFLNnO‐II, DFLNnO I/DFLNO‐II, 5330a, 6400a5, 6400b	6 mo	12, 18 mo	Motor and language skills, socioemotional development, executive function, working memory, ability to stand or walk alone	Positive association between 5311a in HM at 6 mo and motor skills at 18 mo (*p* = 0.003); Positive association between relative abundance of fucosylated (*p* = 0.007) and sialylated HMOs (*p* = 0.033) in HM at 6 mo and language skills at 18 mo; Positive association between FLSTc in HM at 6 mo and motor skills at 18 mo among secretors; Positive association between relative abundance of LSTb in HM at 6 mo and working memory and executive function at 18 mo among children of non‐secretor mothers (*p* = 0.007); Inverse association between 5130c in HM at 6 mo with language skills at 18 mo (*p* = 0.002)
								Inverse association between relative abundance of non‐fucosylated neutral HMOs (*p* < 0.001), LNT (*p* = 0.006), LNnT (*p* = 0.006), LNH (*p* = <0.001), pLNH (*p* = 0.006), IFLNH I (*p* = <0.001), 5300a (*p* = 0.002) in HM at 6 mo and language skills at 18 mo among children of secretor mothers; Inverse association between LSTc in HM at 6 mo and social emotional development at 18 mo among children of secretor mothers (*p* = 0.008); Inverse association between 6’‐SL in HM at 6 mo and walking at 12 mo among of children of secretor mothers (*p* = 0.003)	
Willemsen et al., 2023 [32]	Netherlands	L‐P	Gender not specified (*n* = 67)	2’‐FL, 3‐FL, 6’‐SL, 3’‐SL, DF‐TF‐LNH, DFL, F‐LNH, IF‐LNH‐I, LNDFH‐I, LNDFH‐II, LNFP‐I, LNFP‐II, LNFP‐III, LNFP‐V, LNH, LNnH, pLNH, LNT, LNnT, LST a, LST b, LST c	2, 6, 12 w	3 y	Executive functioning, inhibitory control (motor inhibition, verbal inhibition, impulse control)	Positive associations between 2’‐FL (β = 5.21, 95 % CI: 0.84 – 9.57) and grouped fucosylated HMOs (β = 3.43, 95 % CI: 0.30 – 6.56) in HM at the first 12 w and executive functions at 3 y	Gestational age at birth, maternal educational level, executive functioning of the parent(s), secretor status
**Proteins**									
Jorgensen et al., 2021 [40]	United States	L‐P	Gender not specified (*n* = 659)	Lactalbumin, lactoferrin, osteopontin, IgA	6 mo	12, 18 mo	Motor and language skills, socioemotional development, executive function, working memory, ability to stand or walk alone	Positive association between lactalbumin in HM at 6 mo and motor skills at 18 mo (*p* = 0.038); Inverse association between IgA (*p* = 0.018) and lactoferrin (*p* = 0.044) in HM at 6 mo and motor skills at 18 mo; Inverse association between osteopontin in HM at 6 mo and standing and walking at 12 mo in children of secretor mothers (*p* = 0.007)	Maternal age, maternal height, maternal BMI, parity, maternal education, maternal food security, maternal HIV status, Hb, household assets, residential location, infant sex, season at the time of sample collection
**Carotenoids**									
Zielinska et al., 2019 [14]	Poland	L‐P	Gender not specified (*n* = 53)	β‐carotene, lycopene, lutein, zeaxanthin	1, 3, 6 mo	6 mo	Psychomotor development	Positive association between β‐carotene in HM during the first 3 mo with motor development at 6 mo (β = 0.359; *p* < 0.05); No associations between concentration of Lutein + Zeaxanthin and Lycopene in HM during the first 3 mo and motor development at 6 mo	Parental age, parental education level, marital status, maternal psychological status, maternal nutrition, household income, children in the household, gestational age, birth parameters, gender, child anthropometric parameters, child nutrition

d – days; h – hours; mo – months; wk – weeks; y – years.

ASQ – Ages and Stages Questionnaire; BRW – brain weight; CC – case‐control study; CS – cross‐sectional study; Hb – haemoglobin; HM – human milk; LAZ – length‐for‐age Z‐score; L‐P – longitudinal prospective study; Motor‐2 – Motor Development Tool; OFC – occipitofrontal head circumference; RC – randomized control trial.

**Bioactive components**: 2’‐FL – 2’‐fucosyllactose; 3‐FL – 3‐fucosyllactose; 3’‐SL – 3’‐sialyllactose; 6’‐SL – 6’‐sialyllactose; 4021a, 4100a, 4100b, 4120a, 4211a, 4211b, 4211c, 4240a, 4320a, 5130a, 5130b, 5130c, 5230a, 5230b, 5300a4, 5311a, 5330a, 6400a5, 6400b – no literature name; AA – arachidonic acid; AdA – adrenic acid; ALA – α‐linoleic acid; A‐tetra – alpha‐tetrasaccharide; C16:1 – palmitoleic acid; DeoxyCer – deoxyceramides; DFL – difucosyllactos; DFLac – difucosyllactose; DFLNH – difucosyllacto‐N‐hexaose; DFLNHa – difucosyllacto‐N‐hexaose a; DFLNHb – difucosyllacto‐N‐hexaose b; DFLNHc – difucosyllacto‐N‐hexaose c; DFLNnO I – difucosyllacto‐N‐octaose I; DFLNnO‐II – difucosyllacto‐N‐octaose II; DFLNT – difucosyllacto‐N‐tetrose; DFNH – difucosyllacto‐N‐hexaose; DFpLNH‐II – difucosyl‐para‐lacto‐N‐hexaose II; DGLA – dihomo‐γ‐linolenic acid; DHA – docosahexaenoic acid; DHC – dehydrocholesterol; DPA – docosapentaenoic acid; DSLNH – disialyllacto‐N‐hexaose; DSLNT – disialyllacto‐N‐tetrose; EPA – eicosapentaeonic acid; FAs – fatty acids; FDSLNH – fucodisialyllacto‐N‐hexaose; FLNH – fucosyllacto‐N‐hexaose; F‐LNO – fucosyllacto‐N‐octaose; Fuc – fucosylated; GLA – γ‐linolenic acid; IFLNH‐I – fucosyl‐para‐lacto‐N‐hexaose I; IFLNH‐III – fucosyl‐para‐lacto‐N‐hexaose III; LA – linoleic acid; LDFT – lactodifucotetraose; LDFT – lactodifucotetraose; LNA – α‐linolenic acid; LNDFH‐I – lacto‐N‐difucohexaose I; LNDFH‐II – lacto‐N‐difucohexaose II; LNFP – lacto‐N‐fucopentaose; LNFP‐I – lacto‐N‐fucopentaose I; LNFP‐II – lacto‐N‐fucopentaose II; LNFP‐III – lacto‐N‐fucopentaose III; LNFP‐V – lactose‐N‐fucopentaose V; LNH – lacto‐N‐hexaose; LNT – lacto‐N‐tetraose; LNnH – lacto‐N‐neohexaose; LNnT – lacto‐N‐neotetraose; LNT – lacto‐N‐tetraose; LSTa – sialyllacto‐N‐tetraose a; LSTb – sialyllacto‐N‐tetraose b; LSTc – sialyllacto‐N‐tetraose c; MFLNH‐I – monofucosyllacto‐N‐hexaose I; MFLNH‐III – monofucosyllacto‐N‐hexaose III; MFpLNH‐IV – monofucosyllacto‐N‐hexaose V; MUFA – monounsaturated fatty acids; NA – nervonic acid; OA – oleic acid; PA – palmitic acid; pLNH – para‐lacto‐N‐hexaose; pPEs – plasmaloges; PUFAs – polyunsaturated fatty acids; SFAs – saturated fatty acids; Sia – sialic acid; S‐LNH – sialyl‐lacto‐N‐hexaose; TFLNH – trifucosyllacto‐N‐hexaoese.

### 2.3 Quality Assessment

Methodological quality was assessed using the Joanna Briggs Institute (JBI) tool for observational studies (Table S3), cross‐sectional studies (Table S4), and case‐control studies (Table S5). The tool addressed confounders, statistical analysis, follow‐up, and outcomes [24]. The quality of RCTs was assessed with the Cochrane Risk of Bias (RoB 2) tool [25] (Table S6). Based on the answers to the checklist questions, reviewers judged the risk of bias for each study, categorizing it as low, moderate, or high risk.

## 3 Results

**FIGURE 1:**
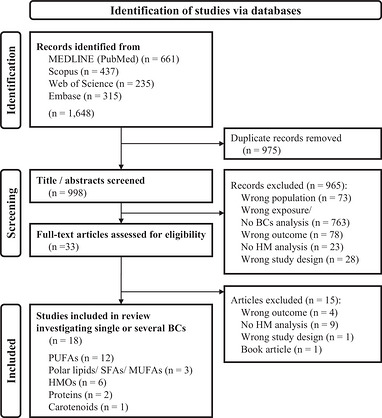
PRISMA flow diagram of literature search and study selection process.

A total of 1648 articles were identified in PubMed, Scopus, Web of Science, and Embase, as shown in the PRISMA flow chart [26] (Figure 1, Table S1). After screening titles and abstracts, 965 articles were excluded mainly due to wrong population (*n* = 73), wrong exposure/no BCs analysis (*n* = 763), or wrong outcome (*n* = 78). 33 articles were then subjected to full‐text screening, and finally, 18 articles were included in the analysis. Of these, 12 analyzed the effects of PUFAs on infants' psychomotor development, while six focused on HMOs, three on polar lipids / SFAs / monounsaturated fatty acids (MUFAs), two on proteins, and one on carotenoids.

### 3.1 Description of Included Studies

Most of the included studies were conducted in high‐income countries, according to the World Bank Country and Lending Groups [27], such as Australia [28], France [29], Italy [30], the Netherlands [31, 32], Spain [33, 34], Sweden [35], Poland [14], and the United States [36, 37, 38, 39, 40, 41, 42] (Table 1). Two studies were conducted in upper‐middle‐income countries; one in Brazil [43] and one in South Africa [44]. The number of mother‐child dyads consisted in total of 3,551 pairs, ranging from small sample sizes (*n* = 19, [35]) to large cohorts (709 dyads, [29]). The publication period ranged from 2000 to 2023. Among the included studies, 14 were cohort studies, one case‐control study, one cross‐sectional, and two RCTs. HM samples were collected at multiple time points, from the first day postpartum up to 12 months. Most samples were obtained at 1 month (8 studies), 3 months (7 studies), or 6 months (8 studies) postpartum. In all studies, mothers received detailed instructions on expressing milk, in accordance with the study's requirements. The procedure was generally standardized, involving either an electronic breast pump or manual expression into sterile plastic cups. The samples were then stored at −20 ° C or −80 ° C. Psychomotor development was assessed using validated tools, with the Ages and Stages Questionnaire (ASQ‐Br; ASQ‐2; ASQ‐3) [29, 41, 43] and Bayley Scales of Infant Development (BSID‐I; BSID‐II) [30, 33, 34] being the most frequently used methods. The assessment time points ranged from 1 month [43] to 12 years [31]. Outcomes were primarily measured at 6 months (7 studies) or 12 months (6 studies). Psychomotor endpoints included hand‐eye coordination, cognitive, linguistic, mental, and motor development (Table 1). The studies adjusted results for various confounding variables, with maternal age being a consistent factor across all. The potential for conducting a meta‐analysis was explored to synthesize findings comprehensively. Heterogeneity was evaluated qualitatively, considering differences in HM collection timing and methods, assessment time points and types of psychomotor measures, and statistical approaches. Due to substantial heterogeneity in assessment timing, measurement methods, sample collection, and statistical adjustments across studies, a meta‐analysis was not considered appropriate. Therefore, the findings were summarized narratively.

### 3.2 Study Quality

The quality of included studies was assessed as described above. The cohort studies [14, 29, 30, 31, 32, 34, 35, 36, 37, 39, 40, 42, 43, 45], the cross‐sectional study [44], and the case‐control study [41], analyzed with the JBI tool, consistently showed low risk of bias and a high quality, except for one study [35], which did not report confounders (Tables S3–S5). The RCTs also showed low risk of bias evaluated with the Cochrane RoB 2 tool, aside from the concern about bias in the selection of reported results (i.e., potential distortion from selectively reporting certain outcomes), and were therefore rated overall as studies with moderate risk of bias [28, 33] (Table S6).

### 3.3 PUFAs

PUFAs were the most frequently measured group of BCs in studies related to infant psychomotor development. In total, 10 studies included 2,376 mother‐child dyads [14, 28, 29, 30, 31, 33, 34, 35, 39, 44], where PUFAs in HM were quantified using fast‐gas chromatography (FAST‐GC), high‐performance liquid chromatography (HPLC), hydrophilic interaction liquid chromatography (HILIC), or gas‐liquid chromatography (GLC). The outcomes were measured at various time points ranging from 1 month to 12 years, with two studies reporting results separately for boys and girls.

**FIGURE 2:**
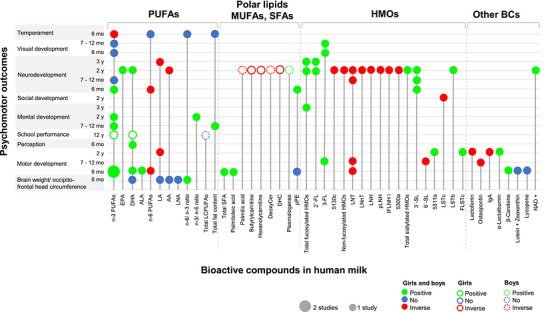
Lollipop diagram showing synthesized results on associations between HM BCs and children's psychomotor development. Circle size indicates the number of studies reporting the same correlation. The “neurodevelopment” outcome includes language, eye‐hand coordination, and executive functions. The “motor development” outcome encompasses standing, walking, and motor skills.

Overall, the levels of n‐3 PUFAs were generally positively associated [14, 28, 30, 31, 34, 35, 39, 44], whereas the levels of n‐6 PUFAs were inversely associated with motor and neurodevelopment [14, 28, 29, 44] (Figure 2). Particularly, the levels of n‐3 PUFAs were positively and the levels of n‐6 PUFAs were inversely associated with locomotor skills and eye‐hand coordination at 6 months [44] (Table 1). Notably, the levels of both n‐3 and n‐6 PUFAs levels were positively associated with mental development at 12 months [30], whereas the levels of n‐3 PUFAs and the ratios of n‐3/n‐6 PUFAs were positively associated with mental development at 14 months [34]. The total n‐3 PUFAs levels were inversely associated with *negative affectivity*, a temperament subscale at 3 months [39]. Another study showed a positive association between n‐3 PUFAs levels and Cito‐test scores, a measure of school performance, at 12 years in girls [31]. One study found that children who received HM from mothers who supplemented fish oil had higher blood plasma n‐3 PUFAs concentrations. However, there were no effects on visual development at 2.5 and 7.5 months or on neurodevelopment at 12 months [33]. Similar results were observed when analyzing individual n‐3 PUFAs levels. DHA levels were positively associated with the perception subscale at 6 months in both boys and girls, and with school performance at 12 years in girls [31]. DHA and α‐Linolenic acid (ALA) levels were positively associated with motor development at 6 months [14]. EPA and DHA levels were positively associated with neurocognitive development – nearly all cognitive outcomes from the Griffiths Mental Development Scales (GMDS) – including hand and eye coordination at 2.5 years [28]. DHA and AA levels showed no association with brain weight (BRW) or occipitofrontal head circumference (OFC) at 1 and 3 months [35], but the AA/DHA ratio was positively associated with BRW and OFC at those ages [35]. No association was observed between the n‐6/n‐3 PUFAs ratio and temperament at 3 months [39], nor between n‐6 PUFAs levels and temperament at that age [39]. Similar findings were observed when analyzing levels of individual n‐6 PUFAs. LA levels were inversely associated with locomotor function, measured with Motor‐2 at 2 years, and neurocognitive development, measured with ASQ‐2 at 3 years [29]. Levels of AA levels were also inversely associated with neurocognitive development, particularly vocabulary skills, at 2.5 years [28]. No significant associations were found between LA or AA levels and brain weight or OFC at 1 and 3 months [35]. Positive associations were identified between HM fat content and mental development at 12 months [30]. No associations were found between total fat of HM and temperament at 3 months [39], or between total LC‐PUFAs and school performance at 12 years in boys [31].

### 3.4 Polar Lipids, SFAs, and MUFAs

The relationship between other FAs, in addition to PUFAs, and psychomotor development in children was analyzed in two studies involving a total of 121 mother‐child dyads [41, 42]. These FAs were mainly quantified using ultra‐HPLC and hydrophilic interaction chromatography (HILIC‐MS). The children ranged in age from 1 month to 2 years.

The following groups of FAs were analyzed in the studies: SFAs, MUFAs, glycerophospholipids, acylcarnitines, and sphingolipids. Overall, limited evidence suggests that plasmalogens, palmitoleic acid (C16:1), and SFAs are positively associated with psychomotor or neurocognitive development, whereas deoxyceramides, hexanoylcarnitine, palmitic acid, butyrylcarnitine, and dehydrocholesterol show inverse associations with developmental outcomes; ethanolamine‐containing plasmalogens (pPEs) show no consistent associations (Figure 2). Specifically, the levels of plasmalogens showed a positive association with neurocognitive development, as measured by ASQ‐2, at the second year of life in girls who are at risk for neurodevelopmental disease (NDD) (Table 1). In the same study, butyrylcarnitine and dehydrocholesterol (DHC) levels demonstrated inverse associations with neurocognitive development, assessed by ASQ‐2, at the second year of life in boys at risk for NDD [41]. Palmitoleic acid (C16:1), a MUFA, and levels of SFAs were positively associated with motor development at 6 months [44]. No associations were found between levels of different pPEs and psychomotor or neurodevelopment at 1 month or 4 months [42]. Levels of other phospholipids such as deoxyceramides (DeoxyCer), hexanoylcarnitine, and palmitic acid (PA) showed inverse association.

### 3.5 HMOs

The second largest group of BCs addressed in the selected studies related to HMOs. These were analyzed across six articles, involving a total of 1,107 mother‐child dyads [32, 36, 37, 40, 43, 45]. HMOs were primarily measured using HPLC, nanoscale liquid chromatography (Nano‐LC‐Chip), high‐performance anion‐exchange chromatography (HPAEC), and liquid chromatography‐fluorescence (LC‐FLD). The studies involved children aged between 1 month and 3 years. All studies investigating the association between HMOs and psychomotor development in children considered the maternal secretor status (secretor or non‐secretor), or the production of fucosylated HMO alpha‐tetrasaccharide (A‐tetra+ and A‐tetra‐). Stratification according to the LEWIS‐status of the mother was not carried out in any study.

Analyses of the fucosylated neutral, non‐fucosylated neutral, and sialylated HMOs were reported. Overall, the strong evidence suggests that specific fucosylated HMOs and specific sialylated HMOs (e.g., 3‐siallactose – 3’‐SL, LSTb, 5311a) are positively associated with neurocognitive, language, and motor development, whereas certain non‐fucosylated neutral HMOs (e.g., LNT, LNnT, LNH, pLNH, IFLNH I, and 5300a), the newly identified HMO 5130c, and specific sialylated HMOs (e.g., 6’‐siallactose – 6’‐SL, and LSTc) show inverse associations with developmental outcomes (Figure 2). Particularly, 2’‐FL levels analyzed in HM collected during the first 3 months showed positive associations with neurocognitive development at 24 months [36] and at 3 years, measured by executive functions [32] (Table 1). 2’‐FL levels at 6 months showed no association with neurocognitive development at 24 months [36]. 3‐FL levels were positively associated with motor development and visual reception in the first year of life. This study also confirms that gut microbiota and HMOs are independently and interactively related to cognitive development [45]. The levels of 5130c were inversely associated with neurocognitive development, specifically language development, at 18 months [40]. Levels of total fucosylated HMOs were positively associated with language development at 18 months among children of secretor mothers [40] and at 3 years among both children of secretor and non‐secretor mothers [32],

Non‐fucosylated neutral HMOs, such as lacto‐*N*‐tetraose (LNT), lacto‐*N*‐neotetraose (LNnT), lacto‐*N*‐hexaose (LNH), p‐lacto‐*N*‐hexaose (pLNH), isofucosyl‐Lacto‐*N*‐hexaose I (IFLNH I), and 5300a, a newly identified non‐fucosylated HMO, were inversely associated with psychomotor development, including emotional and language development [40]. LNT, in particular, was positively associated with the risk of inadequate development in personal‐social skills, motor skills, and personal skills [43], during the first year of life among children of secretor mothers.

The levels of total sialylated HMOs showed a positive association with neurodevelopment, including language at 18 months among children of secretor mothers [40]. Specifically, 5311a, a newly identified sialylated HMO, was positively associated with motor development at 18 months among children of secretor mothers [40]. Furthermore, 3’‐SL levels were positively associated with neurodevelopment, including language abilities in the first two years in children of the A‐tetra+ mothers’ group [37]. Levels of sialyllacto‐*N*‐tetraose b (LSTb) were positively associated with neurodevelopment, including working memory and executive functions at 18 months among children of non‐secretor mothers [40]. However, levels of 6’‐SL were inversely associated with walking at 12 months, while levels of sialyllacto‐*N*‐tetraose (LSTc) were inversely associated with social and emotional development at 18 months among children of secretor mothers [40]. Finally, fucosyl‐sialyllacto‐*N*‐tetraose c (FLSTc) was positively associated with motor development at 18 months among children of secretor mothers [40].

### 3.6 Other BCs

Besides PUFAs, other FAs, and HMOs, three studies examined carotenoids, proteins, hormones, and coenzymes. These BCs were measured using HPLC, HILIC‐MS, and Nano‐LC‐Chip. Overall, 794 mother‐child pairs were analyzed, with children aged 6 to 18 months (Table 1).

Overall, limited evidence suggests that β‐carotene, lactalbumin, and nicotinamide adenine dinucleotide (NAD^+^) are positively associated with psychomotor or neurodevelopmental outcomes, whereas osteopontin, IgA, and lactoferrin show inverse associations with motor development; no associations were found for lycopene or lutein + zeaxanthin (Figure 2). Specifically, β‐carotene levels were positively associated, while no associations between lycopene, lutein + zeaxanthin, and motor development in children at 6 months were observed [14] (Table 1). An inverse association was observed between levels of proteins such as osteopontin, in mothers with secretor status, and their children's motor development at 12 months [40]. Additionally, an inverse association was observed between IgA and lactoferrin levels in mothers with both secretor and non‐secretor status, and their children's motor development at 18 months [40]. A positive association was identified for lactalbumin levels in mothers with either secretor or non‐secretor status and their children's motor development at 18 months (Figure 2) [40]. A positive association were also found between levels of nicotinamide adenine dinucleotide (NAD^+^), a coenzyme, and neurodevelopment, as measured by ASQ‐2, during the second year of life in both boys and girls [41].

## 4 Discussion

This systematic review aimed to synthesize and critically evaluate the available evidence on the associations between individual HM BCs and psychomotor development in children from birth to 18 years of age. The data synthesis of the included studies showed substantial heterogeneity in both the timing and methodology of the HM sample collection. Because concentrations of specific BCs in HM exhibit a circadian variation [46, 47] and are influenced by the stage of lactation [47, 48], the variability in HM sampling time points across studies prevented meta‐analysis. Implementing standardized sampling protocols and clearly defined collection time points will be essential to improve comparability across studies, enhance the reliability of findings on HM BCs, and enable more robust quantitative syntheses in future research.

Another major challenge in synthesizing results arises from the high heterogeneity in the assessment tools used to measure children's psychomotor development. This variability reflects the use of different age‐appropriate instruments designed to ensure valid and reliable assessments across developmental stages and to capture the dynamic nature of early childhood development [49, 50]. Moreover, while some studies mainly focused on motor development, others assessed broader cognitive domains, further increasing the diversity of reported outcomes.

### 4.1 PUFAs

The most frequently studied n‐3 PUFAs were DHA and EPA, which compete as substrates for enzymes and are products of AA metabolism associated with n‐6 PUFAs [51]. Overall, moderate evidence suggests that higher n‐3 PUFA levels and a higher n‐3/n‐6 ratio may be beneficial for psychomotor development [14, 30, 31, 33, 34, 35, 39], whereas higher n‐6 PUFA levels may be associated with less favorable developmental outcomes [28, 35, 39, 44], although study results remain inconsistent. For example, Xiang et al. found a positive correlation between the AA/DHA ratio and brain development (BRW/OFC) [35]. Additionally, an inverse association between n‐3 PUFAs levels and temperament, here called “negative affectivity” at 3 months suggests a potential role for n‐3 PUFAs in early emotional development and regulation [39]. Therefore, lower “negative affectivity” may reflect greater emotional stability, which could be associated with more favorable psychomotor and overall developmental trajectories [52].

Animal studies provide potential mechanistic explanations for the positive effects of n‐3 PUFAs. Thus, experiments in mice suggest that DHA may enhance motor coordination, spatial memory, sensorimotor integration, and cognitive development [53, 54], while EPA supplementation may promote visual and emotional development [54]. Notably, the combination of ARA and DHA in artificial milk fed to mouse pups starting 48 h after birth was capable of supporting normal brain weight development [55]. The underlying mechanisms of n‐3 PUFAs are supposed to involve improved neuronal and brain development, synaptogenesis, synaptic plasticity, myelination, and anti‐inflammatory effects [51, 53, 56, 57, 58, 59, 60]. In contrast, unfavorable effect of n‐6 PUFAs may be related to the induction of pro‐inflammatory eicosanoids, particularly LA, which may impair cognitive development [51, 61]. Maternal diet plays a crucial role in determining HM composition, specifically PUFAs, and is presumed to significantly influence a child's psychomotor and neurological development [62, 63]. Supporting this, animal studies demonstrate that a diet rich in ALA by mother animals during pregnancy and lactation facilitates an increased biosynthesis of DHA in the offspring. This effect may suggest a pathway for greater DHA uptake, with long‐term benefits for memory and brain development [56].

It is important to note that the results of studies in this review indicate gender‐specific effects of n‐3 PUFAs on psychomotor skills [31]. Thus, a positive correlation was found between DHA levels in HM at 6 months after birth and academic performance at 12 months of age in breastfed girls but not in boys. Despite the well‐documented benefits of breastfeeding for cognitive development [1, 64, 65], evidence for gender‐specific differences in these effects remains limited. Supporting this, an animal study showed that female rats had higher DHA levels in their liver and plasma than male rats after ALA supplementation, suggesting enhanced conversion of ALA to DHA in females [66]. This potential mechanism was related to ovarian steroid hormone signaling in females of reproductive age, which may increase endogenous DHA synthesis and serve as an adaptive response to insufficient dietary intake of omega‐3 fatty acids. Since the effects of DHA on cognitive development are well established [57, 58, 67, 68], we hypothesize that higher DHA concentrations in girls may promote better cognitive development through improved conversion of ALA to DHA. The possible mechanism might involve hormonal regulation of PUFA metabolism. Therefore, an animal study reported that female rats have a greater capacity to convert ALA into DHA than males, probably due to higher expression of desaturases in the liver [69]. Further research is needed to clarify the underlying mechanisms behind the gender‐specific effects of HM PUFAs on children's cognitive and psychomotor development later in life, with psychological and social environment considerations as essential confounding factors.

### 4.2 Polar Lipids, SFAs, and MUFAs

The synthesis of findings indicates limited evidence on the associations between the levels of polar lipids, SFAs, and MUFAs in HM on psychomotor development in children [41, 42, 44]. An inverse association between levels of HM sphingolipid deoxyceramides and neurocognitive development was reported in a single study only [41]. The underlying mechanism may involve ceramide‐mediated formation of reactive oxygen species (ROS), thereby increasing oxidative stress [70], a suggestion also supported by studies in mice [71]. In turn, ROS can cause neuronal damage by attacking cellular lipid membranes, particularly in the brain. This disruption can impair signal transmission or lead to apoptosis (cell death) [72]. Conversely, the study by Siziba et al. (2019) found a positive association between levels of SFA and palmitoleic acid, a MUFA, in HM and locomotor development at 6 months. These findings are supported by a recent human study suggesting that polar lipids, particularly sphingolipids, may support the neurobehavioral development of very low birth weight infants when shortages are addressed with sphingomyelin‐fortified milk [73]. Notably, similar to findings for HM PUFAs, gender‐specific effects of various glycerophospholipids and sphingolipids in neurocognitive development during the second year of life were reported [41]. A possible mechanism involves estrogen‐mediated antioxidant properties in females, which may help explain why girls are better protected against oxidative stress [74]. Further well‐designed studies are needed to strengthen the evidence and clarify the associations between HM polar lipids, SFAs, and MUFAs, and psychomotor development.

### 4.3 HMOs

HMOs comprise a large group of BCs that were investigated in the included studies, with strong evidence indicating positive associations with psychomotor development depending on their fucosylation or sialylation status. The levels of neutral fucosylated, neutral non‐fucosylated, and sialylated acidic HMOs [12, 13, 46] are partly determined by maternal secretor and Lewis statuses. Mothers carrying the Se(+)‐gene (secretors) express the enzyme α1‐2‐fucosyltransferase (FUT2), which is responsible for the synthesis of α1,2‐fucosylated HMOs, such as 2’‐FL [17, 75, 76, 77, 78, 79]. Non‐secretor mothers, with inactivated FUT2 and increased FUT3 activity, have low or undetectable levels of fucosylated HMOs in HM [17, 78]. Similarly, Lewis‐positive mothers who carry the Le(+)‐gene express α1,3/4‐fucosyltransferase (FUT3), leading to the production of α1,3/4‐fucosylated HMOs such as LNFP II. Conversely, Lewis‐ and secretor‐negative mothers have lower levels of these fucosylated HMOs [17, 75, 76, 77, 78]. These variations emphasize the need to adjust results based on maternal secretor status. Supporting this, a positive association between the levels of fucosylated and sialylated HMOs in the milk of secretor‐status mothers and language development [40], while no associations were found when children of both secretor and non‐secretor mothers were analyzed together. Correctly determining maternal secretor status is essential for future studies. When studies identified non‐secretors, they used cutoffs such as “> 6 % relative abundance of α1–2‐linked fucosylated HMOs” [40] or “presence or near absence of 2’‐FL and LNFP I” [43], which can lead to inconsistencies. Future research must adopt standardized cutoff criteria, as HM from nonsecretor mothers might contain small amounts of 2’‐FL and risk misclassification as secretors [17, 78]. As seen in studies by Cho et al. (2021, 2023) [37, 38], stratification by secretor status was not possible due to unequal sample sizes [37, 45], so the groups were classified as A‐tetra+ and A‐tetra‐. A‐tetra is an HMO present only in the HM of secretor mothers with blood group A or AB [80]. Since A‐tetra is believed to be important for producing sialylated HMOs, results showing stratification based on A‐tetra+ suggest stronger positive associations between 3’‐SL levels and language development in 12 month‐old children, especially receptive and expressive language skills [37]. Moreover, stratification by A‐tetra+ HM revealed positive links between 3‐FL and visual, gross motor skills, and gut *B. bifidum* [45].

Evidence regarding the relationship between sialylated HMOs and psychomotor development is very limited and has been examined in only one study [40]. Interestingly, although sialylated HMOs are present in the HM of both secretor and non‐secretor mothers, positive associations with working memory and executive function, particularly for LSTb levels were observed only in infants of non‐secretor mothers. Supporting this, a cohort study found higher LSTb levels in the HM of non‐secretor mothers compared to secretor mothers [46]. Overall, these findings suggest a possible beneficial role of sialylated HMOs in psychomotor development. The underlying mechanisms may involve sialic acid (SA), a key component of sialylated HMOs that supports neural function and synaptogenesis [81], and plays an essential role in neuronal transmission, thereby supporting cognitive development [82]. Interactions between HMOs and gut bacteria, particularly *Bifidobacteria*, were demonstrated in a study by Cho et al. (2023) [38], indicating a potential link between HMOs and the gut microbiota that could promote positive cognitive outcomes. One proposed mechanism involves bacterial sialidase, particularly BbSia2 from *Bifidobacterium bifidum*, which facilitates the release of SA [83]. The beneficial role of sialylated HMOs in neurodevelopment is further reinforced by animal studies showing that supplementation with 3’‐SL and 6’‐SL improves cognitive function, likely through beneficial modulation of the gut microbiota [84, 85, 86, 87]. Another mouse study supports these findings, showing that 6’‐SL plays a favorable role in neurodevelopment. In this research, lack of 6’‐SL during lactation was associated with downregulation of genes involved in neuronal circuit formation and patterning, lower levels of circulating serotonin, kynurenate, and picolinate, and changes in gut microbiota composition [88]. Notably, an inverse relationship was observed between 6’‐SL and LSTc levels in the human milk of secretor mothers at 6 months and subsequent developmental outcomes, including walking at 12 months and social‐emotional development at 18 months in their children [40]. One possible explanation for this finding is the timing of milk collection, which was not recorded in the study. Research shows that concentrations of both 6’‐SL and LSTc fluctuate throughout the day in secretor mothers, with higher levels at 1:00 and 6:00 a.m compared to the afternoon [46]. This underscores the importance of standardized sample collection times in studies examining HMO composition.

Among other individual HMOs, moderate evidence suggests that higher levels of 2’‐FL in HM are positively associated with infant cognitive development at 2 and 3 years of age [32, 36]. These findings are supported by studies in rodents and piglets, which demonstrated that 2’‐FL might enhance cognitive development, possibly through modulation of synaptic plasticity [20, 21, 86]. Since 2’‐FL concentrations decrease over lactation and exhibit diurnal variation [46], the timing of milk collection could influence study results.

### 4.4 Proteins, Carotenoids, Hormones, and Proteins

Proteins and hormones, such as α‐lactalbumin, lactoferrin, and sIgA, are important BCs in HM, are important BCs in HM. Their concentrations are highest in colostrum and decrease in transitional and mature milk, reflecting their key role in early immune system maturation [48, 89, 90, 91, 92]. However, evidence about their associations with psychomotor and neurodevelopmental outcomes remains limited and, in some cases, contradictory. Positive associations have been reported for β‐carotene, α‐lactalbumin, and NAD^+^, whereas inverse associations have been observed for osteopontin, IgA, and lactoferrin in relation to motor development [14, 40]. One possible explanation for these inconsistent findings is the influence of maternal and infant health status. For example, infections during the wet season may increase lactoferrin concentrations in HM, potentially confounding links to motor development. Supporting this, lactoferrin and IgA levels have been found to be elevated in the milk of mothers whose infants experienced gastrointestinal or respiratory illnesses compared to healthy infants [93]. Conversely, studies in preterm infants have shown that higher lactoferrin levels in HM are associated with increased overall brain volume, even after adjusting for key confounders [94]. Moreover, lactoferrin supplementation in piglets has been shown to improve cognitive performance and working memory [95]. The underlying mechanism may involve upregulation of brain‐derived neurotrophic factor (BDNF) and increased phosphorylation of cAMP response element‐binding protein (CREB), both critical for neurodevelopment [95]. Similarly, animal studies support beneficial effects of α‐lactalbumin supplementation, including improved motor function and modest improvements in gut and cognitive development in preterm pigs [96]. These benefits may be mediated by increased expression of polysialic acid, a marker of neuroplasticity involved in cell migration, progenitor cell differentiation, and axonal growth. Additional mechanisms may involve structural brain development, tryptophan metabolism, and serotonin synthesis [97]. On the other hand, findings regarding osteopontin and sIgA remain inconsistent. Jørgensen et al. (2021) reported inverse associations between osteopontin and motor milestones, as well as between sIgA levels at 6 months and motor development at 18 months [40]. However, animal studies do not support these findings. For instance, osteopontin‐enriched diets in young pigs have been associated with increased brain region volumes and enhanced exploratory behavior [98]. The proposed mechanism involves increased expression of myelination‐related proteins and enhanced proliferation and differentiation of oligodendrocyte precursor cells, along with activation of ERK1/2 and PI3K/Akt signaling pathways [99]. Similarly, other human studies have observed positive associations between sIgA and neurodevelopment, possibly through modulation of the gut–brain axis [100]. Regarding carotenoids, β‐carotene has been positively linked to motor development at 6 months, consistent with its antioxidant and anti‐inflammatory properties and its role in visual and cognitive functions [101]. Its neuroprotective effects are further supported by animal studies demonstrating protection following ischemic brain injury [102]. Notably, some of the inverse associations seen for IgA and lactoferrin were based on analyses that did not account for maternal secretor status [40]. Given the known effect of secretor status on HM composition, future studies should include stratified analyses to improve interpretation and better understand potential interactions among bioactive components in HM.

Growing evidence indicates that HM bioactive compounds function within an integrated and interdependent system rather than in isolation, emphasizing the need to consider potential synergistic interactions. Specifically, interactions between HMOs and polyunsaturated fatty acids (PUFAs) have been suggested [103]. HMOs are known to modulate the gut microbiota and increase the availability of key metabolites such as SA, which are crucial for neurodevelopment, while PUFAs, particularly DHA and ARA, play vital structural and functional roles in brain development. Although direct evidence of synergy between these components is limited, recent cohort studies that analyze HMOs and fatty acids together suggest their associations with infant developmental outcomes may be interdependent rather than independent, implying a coordinated biological effect [103]. The underlying mechanism might involve HMOs’ role in affecting the bioavailability and metabolic utilization of PUFAs through microbiota‐mediated pathways, while PUFAs may modulate neuronal responsiveness to HMO‐derived metabolites, jointly supporting optimal neurodevelopment. Overall, these findings promote a shift from single‐nutrient approaches toward a systems‐level understanding of HM, where interactions between HMOs and PUFAs may play a critical role in shaping developmental outcomes. To better understand the mechanisms and the synergistic effects of HM FAs, HMOs, and neurodevelopment, future studies could employ advanced methods such as machine learning or clustering approaches.

### 4.5 Strengths and Limitations

This systematic review is strengthened by several methodological features that enhance the credibility and robustness of its findings. The involvement of an interdisciplinary team with expertise in neuroscience, HM research, and early life programming allowed for a comprehensive and contextually informed interpretation of the evidence, thereby enhancing the scientific validity of the conclusions. The review was conducted following the PRISMA guidelines, ensuring a transparent, systematic, and reproducible process for study selection and reporting, which strengthens confidence in the methodology's integrity of the methodology. Furthermore, the methodological quality and risk of bias of the included studies were systematically assessed using validated tools, including JBI and the Cochrane RoB instruments. Using these standardized and widely accepted frameworks increases the reliability, consistency, and clarity of the results, supporting the overall credibility of the review.

This systematic review has several limitations. With regard to the included studies, there was considerable heterogeneity in follow‐up periods, timing of HM collection, and the measurement instruments used to assess developmental outcomes, which may limit comparability across studies. In addition, many studies did not account for important confounding factors, such as maternal secretor status, and broader social and psychological influences, as described in Engel's biopsychosocial model [104], potentially affecting the interpretation of the findings. Residual confounding by socioeconomic status, parental cognitive ability, home environment, and caregiving practices remains a concern and should be explicitly addressed in future research. Standardized protocols for HM collection, storage, and analysis, along with more comprehensive adjustment for relevant confounders, are needed to strengthen the evidence base. Limitations of the review process include restriction to English‐language studies and the exclusion of non‐peer‐reviewed sources (e.g., theses, reports, and preprints), which may introduce publication bias, as studies with significant results are more likely to be published.

## 5 Conclusion and Future Prospects

This systematic review indicates that HM BCs may support psychomotor development; however, the strength of evidence varies across different components. The most consistent evidence was found for PUFAs, with n‐3 PUFAs, particularly DHA, generally showing beneficial associations, while higher n‐6 PUFAs were more often associated with less favorable outcomes, though findings remain partly inconsistent. Among HMOs, fucosylated HMOs demonstrated the most consistent positive associations with cognitive development, whereas evidence for non‐fucosylated and sialylated HMOs remains limited and heterogeneous, underscoring the importance of maternal secretor status. For proteins and carotenoids, findings were inconsistent and insufficient to draw firm conclusions. Overall, the current evidence supports a potential role of HM BCs in child neurodevelopment but does not yet allow definitive causal inferences. Broader implications for long‐term developmental or societal outcomes should therefore be interpreted with caution.

Future research should focus on standardizing methods and improving study design. Specifically, it should include standardized protocols for HM sampling (covering timing and lactation stage), harmonized and age‐appropriate neurodevelopmental assessment tools, and longer follow‐up periods to improve comparability across studies. Additionally, more comprehensive adjustment for confounding factors, including maternal secretor status and psychosocial variables such as socioeconomic status, parental cognition, home environment, and caregiving practices is crucial. Finally, integrative analytical approaches, including multi‐component and systems‐level analyses, could help better understand interactions between HM bioactive components and clarify their combined effects on child development.

## Author Contributions

**L. H**.: Conceptualization, methodology, title/abstract/full text article screening, assessment of studies quality, data extraction, data synthesis, writing – original draft, visualization, data interpretation. **E. V**.: Conceptualization, methodology, data extraction, review and editing of final manuscript. **R. E**.: Conceptualization, review and editing of final manuscript. **D. G**.: Conceptualization, project administration, supervision, title/abstract/full text article screening, assessment of studies quality, data extraction, data synthesis, review and editing of original draft and final manuscript.

## Availability of Data and Codes

Data used for the data synthesis are available at the Institut of Child Nutrition, Max Rubner‐Institut and can be requested from D.G.

## Code Availability Statement

The R scripts used for generating the figures and conducting the meta‐analyses included in the Supplementary material.

## Conflicts of Interest

The authors declare no conflict of interest.

## Supporting information




**Supporting File**: mnfr70553‐sup‐0001‐SuppMat.docx.

## Data Availability

The data that supports the findings of this study are available in the supplementary material of this article
