# Supplementary material for: Human Milk Bioactive Compounds and Psychomotor Outcomes in Infants and Children: A Systematic Review
Source: Mol Nutr Food Res. 2026 Jul 23;70(14):e70553. doi: 10.1002/mnfr.70553 (PMC13392589; doi:10.1002/mnfr.70553)
Supplement: Supplementary file 1 — Supporting File: mnfr70553‐sup‐0001‐SuppMat.docx. [file MNFR-70-e70553-s001.docx]

**Supplementary material**

**Human milk bioactive compounds and psychomotor outcomes in** **infants and children: a systematic review**

Lara Huber (1), Eduard Flores Ventura (2), Regina Ensenauer (3), Daria Guseva* (1)

(1) Department of Child Nutrition, Max Rubner-Institut – Federal Research Institute of Nutrition and Food, Karlsruhe, Germany

(2) Department of Biotechnology, Institute of Agrochemistry and Food Technology – Spanish National Research Council (IATA-CSIC), Valencia, Spain

(3) Executive body National Breastfeeding Committee, Max Rubner-Institut – Federal Research Institute of Nutrition and Food, Karlsruhe, Germany

Table S1: Generic search strings based on PECOTS framework approach

| **Generic search strings** | "infant*" OR "child*" OR "baby" OR "infancy" OR "toddler*" OR "newborn" OR "new-born" OR "new born" OR "offspring" OR "mother infant*" OR "mother* child" OR "mother-infant" OR "maternal infant*" OR "maternal child" OR "mother-to-infant" OR "mother to child" OR "neonatal" OR "teenage*" OR "adolescent" OR "pube*"  **AND**  "breastmilk" OR "breast milk" OR "Breastmilk" OR "human milk" OR "mother* milk" OR "maternal milk" OR "breastfeed*" OR "breastfeeding" OR "breastfe*" OR "lactation" OR "colostrum" OR "mother* own milk  **AND**  "bioactive*" OR "bioactive component" OR "bioactive compound" OR "component*" OR "compound*" OR "composition"  **AND**  "psychomotor*" OR "psychomotor development" OR "motor*" OR "motor development" OR "neurodevelopment*" OR "cognition" OR "psychomotor performance" OR "cognitive function*" OR "executive function*" OR "temperament" OR "communication*" OR "child* development" OR "brain development" OR "mental" OR "mental development" OR "intelligence*"  **AND NOT**  "review" OR "systematic review" OR "preterm" OR "mice" OR "rats" OR "rabbit*" OR "very low birth weight" |
| --- | --- |

Table S2: Search strings adapted to databases and number of hits

| **Search database** | **Search strings** | **Number of hits** |
| --- | --- | --- |
| **MEDLINE (Pubmed)** | ("infant*" OR "child*" OR "baby" OR "infancy" OR "toddler*" OR "newborn" OR "new-born" OR “new born”[tiab:~10] OR "offspring" OR "mother infant*"[tiab:~10] OR "mother* child"[tiab:~10] OR "mother-infant" OR "maternal infant*" [tiab:~10] OR "maternal child"[tiab:~10] OR "mother-to-infant" OR "mother to child" [tiab:~10] OR "neonatal" OR "teenage*" OR "adolescent" OR "pube*") AND ("breastmilk" OR "breast milk" [tiab:~10] OR "Breastmilk" OR "human milk" [tiab:~10] OR "mother´s milk" [tiab:~10] OR "mothers milk" [tiab:~10] OR "maternal milk" [tiab:~10] OR "breastfeed*" OR "breastfeeding" OR "breastfe*" OR “lactation” OR "colostrum" OR "mother* own milk"[tiab:~10]) AND ("bioactive*" OR "bioactive component" [tiab:~10] OR "bioactive compound" [tiab:~10] OR "component*" OR "compound*" OR "composition") AND ("psychomotor*" OR "psychomotor development" [tiab:~10] OR "motor*" OR "motor development" [tiab:~10] OR “neurodevelopment*” OR "cognition" OR "psychomotor performance"[tiab:~10] OR "cognitive function*"[tiab:~10] OR "executive function*"[tiab:~10] OR "temperament" OR "communication*" OR "child* development" OR "brain development" [tiab:~10] OR "mental" OR "mental development" [tiab:~10] OR "intelligence*") NOT ("review" [pt] OR "systematic review" [pt] OR "preterm" OR "mice" OR "rats" OR "rabbit*" OR "very low birth weight") | **661** |
| **Scopus** | TITLE-ABS-KEY ("infant*" OR "child*" OR "baby" OR "infancy" OR "toddler*" OR "newborn" OR "new-born" OR "new W/10 born" OR "offspring" OR "mother W/10 infant*" OR "mother* W/10 child" OR "mother-infant" OR "maternal W/10 infant*" OR "maternal W/10 child" OR "mother-to-infant" OR "mother to child" OR "neonatal" OR "teenage*" OR "adolescent" OR "pube*") AND TITLE-ABS-KEY ("breastmilk" OR "breast W/10 milk" OR "human W/10 milk" OR "mother's W/10 milk" OR "mothers W/10 milk" OR "maternal W/10 milk" OR "breastfeed*" OR "lactation" OR "colostrum" OR "mother's own milk") AND TITLE-ABS-KEY ("bioactive*" OR "bioactive W/10 component" OR "bioactive W/10 compound" OR "component*" OR "compound*" OR "composition") AND TITLE-ABS-KEY ("psychomotor*" OR "psychomotor W/10 development" OR "motor*" OR "motor W/10 development" OR "neurodevelopment*" OR "cognition" OR "psychomotor W/10 performance" OR "cognitive W/5 function*" OR "executive W/5 function*" OR "temperament" OR "communication*" OR "child W/5 development" OR "brain W/5 development" OR "mental" OR "mental W/5 development" OR "intelligence*") AND NOT INDEXTERMS ("review" OR "systematic review") AND NOT TITLE-ABS-KEY ("preterm" OR "mice" OR "rats" OR "rabbit*" OR "very low birth weight") | **437** |
| **Web of Science** | TS=(("infant*" OR "child*" OR "baby" OR "infancy" OR "toddler*" OR "newborn" OR "new-born" OR ("new NEAR/10 born") OR "offspring" OR ("mother NEAR/10 infant*") OR ("mother* NEAR/10 child") OR "mother-infant" OR ("maternal NEAR/10 infant*") OR ("maternal NEAR/10 child") OR "mother-to-infant" OR "mother to child" OR "neonatal" OR "teenage*" OR "adolescent" OR "pube*") AND ("breastmilk" OR ("breast NEAR/10 milk") OR ("human NEAR/10 milk") OR ("mother's NEAR/10 milk") OR ("mothers NEAR/10 milk") OR ("maternal NEAR/10 milk") OR "breastfeed*" OR "lactation" OR "colostrum" OR "mother's own milk") AND ("bioactive*" OR ("bioactive NEAR/10 component") OR ("bioactive NEAR/10 compound") OR "component*" OR "compound*" OR "composition") AND ("psychomotor*" OR ("psychomotor NEAR/10 development") OR "motor*" OR ("motor NEAR/10 development") OR "neurodevelopment*" OR "cognition" OR ("psychomotor NEAR/10 performance") OR ("cognitive NEAR/10 function*") OR ("executive NEAR/10 function*") OR "temperament" OR "communication*" OR ("child NEAR/10 development") OR ("brain NEAR/10 development") OR "mental" OR ("mental NEAR/10 development") OR "intelligence*")) NOT TS= ("review" OR "systematic review" OR "preterm" OR "mice" OR "rats" OR "rabbit*" OR "very low birth weight") | **235** |
| **Embase** | ('infant*':ti,ab,kw OR 'child*':ti,ab,kw OR 'baby':ti,ab,kw OR 'infancy':ti,ab,kw OR 'toddler*':ti,ab,kw OR 'newborn':ti,ab,kw OR 'new-born':ti,ab,kw OR ('new NEAR/10 born'):ti,ab,kw OR 'offspring':ti,ab,kw OR ('mother NEAR/10 infant*'):ti,ab,kw OR ('mother* NEAR/10 child'):ti,ab,kw OR 'mother-infant':ti,ab,kw OR ('maternal NEAR/10 infant*'):ti,ab,kw OR ('maternal NEAR/10 child'):ti,ab,kw OR 'mother-to-infant':ti,ab,kw OR 'mother to child':ti,ab,kw OR 'neonatal':ti,ab,kw OR 'teenage*':ti,ab,kw OR 'adolescent':ti,ab,kw OR 'pube*':ti,ab,kw) AND ('breastmilk':ti,ab,kw OR ('breast NEAR/10 milk'):ti,ab,kw OR ('human NEAR/10 milk'):ti,ab,kw OR ('mother* NEAR/10 milk'):ti,ab,kw OR ('mothers NEAR/10 milk'):ti,ab,kw OR ('maternal NEAR/10 milk'):ti,ab,kw OR 'breastfeed*':ti,ab,kw OR 'lactation':ti,ab,kw OR 'colostrum':ti,ab,kw OR 'mother* own milk'):ti,ab,kw AND ('bioactive*':ti,ab,kw OR ('bioactive NEAR/10 component'):ti,ab,kw OR ('bioactive NEAR/10 compound'):ti,ab,kw OR 'component*':ti,ab,kw OR 'compound*':ti,ab,kw OR 'composition':ti,ab,kw) AND ('psychomotor*':ti,ab,kw OR ('psychomotor NEAR/10 development'):ti,ab,kw OR 'motor*':ti,ab,kw OR ('motor NEAR/10 development'):ti,ab,kw OR 'neurodevelopment*':ti,ab,kw OR 'cognition':ti,ab,kw OR ('psychomotor NEAR/10 performance'):ti,ab,kw OR ('cognitive NEAR/10 function*'):ti,ab,kw OR 'executive function*':ti,ab,kw OR 'temperament':ti,ab,kw OR 'communication*':ti,ab,kw OR 'child development':ti,ab,kw OR ('brain NEAR/10 development'):ti,ab,kw OR 'mental':ti,ab,kw OR ('mental NEAR/10 development'):ti,ab,kw OR 'intelligence*':ti,ab,kw) NOT ([review]/lim OR [systematic review]/lim) NOT ('preterm':ti,ab,kw OR 'mice':ti,ab,kw OR 'rats':ti,ab,kw OR 'rabbit*':ti,ab,kw OR 'very low birth weight':ti,ab,kw) | **315** |

Table S3: JBI Risk of Bias assessment cohort studies

| **Checklist for cohort studies** | | | | | | | | | | | | |
| --- | --- | --- | --- | --- | --- | --- | --- | --- | --- | --- | --- | --- |
| **Reference** | **A** | **B** | **C** | **D** | **E** | **F** | **G** | **H** | **I** | **J** | **K** | **Overall** |
| **Hahn-Holbrook et al.,**  **2019** | yes | yes | yes | yes | yes | yes | yes | yes | yes | yes | yes | low |
| **Ferreira et al.,**  **2021** | yes | yes | yes | yes | yes | yes | yes | yes | yes | yes | yes | low |
| **Guxens et al.,**  **2011** | yes | yes | yes | yes | yes | yes | yes | yes | yes | yes | yes | low |
| **Ramadurai et al.,**  **2022** | yes | yes | yes | yes | yes | yes | yes | yes | yes | yes | yes | low |
| **Xiang et al.,**  **2000** | yes | yes | yes | yes | no | yes | yes | yes | yes | yes | yes | moderate |
| **Zielinska et al.,**  **2019** | yes | yes | yes | yes | yes | yes | yes | yes | yes | yes | yes | low |
| **Jorgensen et al.,**  **2021** | yes | yes | yes | yes | yes | yes | yes | yes | yes | yes | yes | low |
| **Willemsen et al.,**  **2023** | yes | yes | yes | yes | yes | yes | yes | yes | yes | yes | yes | low |
| **Bernard et al.,**  **2015** | yes | yes | yes | yes | yes | yes | yes | yes | yes | yes | yes | low |
| **Berger et al.,**  **2020** | yes | yes | yes | yes | yes | yes | yes | yes | yes | yes | yes | low |
| **Dalmeijer et al.,**  **2015** | yes | yes | yes | yes | yes | yes | yes | yes | yes | yes | yes | low |
| **Cho et al.,**  **2021** | yes | yes | yes | yes | yes | yes | yes | yes | yes | yes | yes | low |
| **Cho et al.,**  **2023** | yes | yes | yes | yes | yes | yes | yes | yes | yes | yes | yes | low |
| **Agostoni et al.,**  **2001** | yes | yes | yes | yes | yes | yes | yes | yes | yes | yes | yes | low |

A: Were the two groups similar and recruited from the same population?

B: Were the exposures measured similarly to assign people to both exposed and unexposed groups?

C: Was the exposure measured in a valid and reliable way?

D: Were confounding factors identified?

E: Were strategies to deal with confounding factors stated?

F: Were the groups/participants free of the outcome at the start of the study (or at the moment of exposure)?

G: Were the outcomes measured in a valid and reliable way?

H: Was the follow up time reported and sufficient to be long enough for outcomes to occur?

I: Was follow up complete, and if not, were the reasons to loss to follow up described and explored?

J: Were strategies to address incomplete follow up utilized?

K: Was appropriate statistical analysis used?

Table S4: JBI tool Risk of Bias for cross-sectional studies

| **Checklist for cross-sectional studies** | | | | | | | | | | | |
| --- | --- | --- | --- | --- | --- | --- | --- | --- | --- | --- | --- |
| **Reference** | **A** | **B** | **C** | **D** | **E** | **F** | **G** | **H** | **I** | **J** | **Overall** |
| **Sibiza et al.,**  **2019** | yes | yes | yes | yes | yes | yes | yes | yes | yes | yes | low |

A: Were the groups comparable other than the presence of disease in cases or the absence of disease in controls?

B: Were cases and controls matched appropriately?

C: Were the same criteria used for identification of cases and controls?

D: Was exposure measured in a standard, valid and reliable way?

E: Was exposure measured in the same way for cases and controls?

F: Were confounding factors identified?

G: Were strategies to deal with confounding factors stated?

H: Were outcomes assessed in a standard, valid and reliable way for cases and controls?

I: Was the exposure period of interest long enough to be meaningful?

J: Was appropriate statistical analysis used?

Table S5: JBI Risk of Bias assessment case-control studies

| **Checklist for case-control studies** | | | | | | | | | |
| --- | --- | --- | --- | --- | --- | --- | --- | --- | --- |
| **Reference** | **A** | **B** | **C** | **D** | **E** | **F** | **G** | **H** | **Overall** |
| **Li et al.,**  **2021** | yes | yes | yes | yes | yes | yes | yes | yes | low |

A: Were the criteria for inclusion in the sample clearly defined?

B: Were the study subjects and the setting described in detail?

C: Was the exposure measured in a valid and reliable way?

D: Were objective, standard criteria used for measurement of the condition?

E: Were confounding factors identified?

F: Were strategies to deal with confounding factors stated?

G: Were the outcomes measured in a valid and reliable way?

H: Was appropriate statistical analysis used?

Table S6: RoB2 Risk of Bias assessment RCTs

| **Checklist for RCTs** | | | | | | |
| --- | --- | --- | --- | --- | --- | --- |
| **Reference** | **A** | **B** | **C** | **D** | **E** | **Overall** |
| **Dunstan et al., 2007** | + | + | + | + | - | some concerns |
| **Hurtado et al., 2015** | + | + | + | + | - | some concerns |

A: Bias arising from randomization process

B: Bias due to deviations from intended intervention

C: Bias due to missing outcome data.

D: Bias in measurement of the outcome.

E: Bias in selection of the reported result.

**Supplementary – R script**

Lollipop diagram was created using R and completed with PowerPoint

# Load required libraries

library(ggplot2)

library(tidyr)

library(readxl)

library(dplyr)

# Load Excel file (provide the correct path to your file) data <- read_excel("/Users/larahuber/Masterthesis/R_lollipopdiagram.xlsx", sheet = "Tabelle1")

# Display column names to ensure that selecting the right one

colnames(data)

# Save the name of the first column

bioactive_components <- colnames(data)[1]

# First, remove non-numeric values from the numeric columns and convert them to numeric data_clean <- data %>%

mutate(across(everything(), ~ as.numeric(as.character(.)), .names = "clean_(Zuurveld et al.)"))

# Reshape the data into long format using pivot_longer, keeping the first column (bioactive components) as the category

long_data <- pivot_longer(data, cols = -all_of(bioactive_components), names_to = "Outcome", values_to = "Value")

# Remove rows where the value is 4

long_data <- long_data %>% filter(Value != 4)

# Remove missing values (to retain only relevant entries)

long_data <- long_data[!is.na(long_data$Value), ]

# Set bioactive components as factors to maintain the order from the Excel sheet long_data[[bioactive_components]] <- factor(long_data[[bioactive_components]], levels = unique(data[[bioactive_components]]))

# Set outcomes as factors to maintain the order from the original column names in the Excel sheet long_data$Outcome <- factor(long_data$Outcome, levels = colnames(data)[-1])

# '-1' to exclude the first column (bioactive components)

# Create the lollipop chart

ggplot(long_data, aes(x = .data[[bioactive_components]], y = Outcome)) +

# Add lollipop lines

geom_segment(aes(x = .data[[bioactive_components]], xend = .data[[bioactive_components]], y = Outcome, yend = 0),

color = "grey", size = 1.0, show.legend = FALSE) + # Disable legend for lines

# Add circles for the lollipops

geom_point(aes(fill = factor(Value), size = factor(Value)), shape = 21, stroke = 0, show.legend = FALSE) + # Disable legend for points

labs(title = "Results bioactive compounds and psychomotor development in studies",

x = "Bioactive Compounds",

y = "Outcomes") +

theme_minimal() +

theme(

panel.background = element_blank(),

plot.background = element_blank(),

axis.title = element_text(color = "black"),

axis.text = element_text(color = "black"),

axis.text.x = element_text(angle = 90, hjust = 1),

# Disable vertical gridlines

panel.grid.major.x = element_blank(),

panel.grid.minor.x = element_blank(),

# Keep horizontal gridlines for checkered effect

panel.grid.major.y = element_line(color = "lightgrey", size = 0.5),

panel.grid.minor.y = element_line(color = "lightgrey", size = 0.25),

plot.title = element_text(hjust = 0.5, margin = margin(b = 20), face = "bold")

) +

coord_fixed() + # Ensure square grid

# Keep colors consistent but remove legend

scale_fill_manual(

values = c("0" = "blue", "1" = "red", "2" = "green", "3" = "green"),

guide = "none" # No legend

) +

# Keep sizes consistent but remove legend

scale_size_manual(

values = c("0" = 5, "1" = 5, "2" = 5, "3" = 8),

guide = "none" # No legend

)

Table S7: Reference table R Lollipop diagram

|  | **BRW/OFC first 6 mo** | **Motor develop.ment first 6 mo** | **Motor development 7 mo to 12 mo** | **Motor development 2. y of life** | **Perception first 6 mo** | **School performance 12 y** | **Mental development 7 mo to 12 mo** | **Mental development 2. y of life** | **Mental development 3. y of life** | **Social development 2. y of life** | **Neuro-development first 6 mo** | **Neuro-development 7 mo to 12 mo** | **Neuro-development 2. y of life** | **Neuro-development 3. y of life** | **Visual development first 6 mo** | **Visual development 7 mo to 12 mo** | **Temperament first 6 mo** |
| --- | --- | --- | --- | --- | --- | --- | --- | --- | --- | --- | --- | --- | --- | --- | --- | --- | --- |
| **n-3 PUFAs** | 4 | 3 | 4 | 4 | 4 | 7 | 2 | 2 | 4 | 4 | 2 | 0 | 4 | 4 | 0 | 0 | 1 |
| **EPA** | 4 | 4 | 4 | 4 | 4 | 4 | 4 | 4 | 4 | 4 | 4 | 4 | 2 | 4 | 4 | 4 | 4 |
| **DHA** | 0 | 2 | 4 | 4 | 2 | 7 | 4 | 4 | 4 | 4 | 4 | 4 | 2 | 4 | 4 | 4 | 4 |
| **ALA** | 4 | 2 | 4 | 4 | 4 | 4 | 4 | 4 | 4 | 4 | 4 | 4 | 4 | 4 | 4 | 4 | 4 |
| **n-6 PUFAs** | 4 | 1 | 4 | 4 | 4 | 4 | 4 | 4 | 4 | 4 | 1 | 4 | 4 | 4 | 4 | 4 | 0 |
| **LA** | 0 | 4 | 4 | 1 | 4 | 4 | 4 | 4 | 4 | 4 | 4 | 4 | 4 | 1 | 4 | 4 | 4 |
| **AA** | 0 | 4 | 4 | 4 | 4 | 4 | 4 | 4 | 4 | 4 | 4 | 4 | 1 | 4 | 4 | 4 | 4 |
| **LNA** | 0 | 4 | 4 | 4 | 4 | 4 | 4 | 4 | 4 | 4 | 4 | 4 | 4 | 4 | 4 | 4 | 4 |
| **n-3/n-6 ratio** | 2 | 2 | 4 | 4 | 4 | 4 | 4 | 2 | 4 | 4 | 4 | 4 | 4 | 4 | 4 | 4 | 0 |
| **LCPUFAs** | 4 | 4 | 4 | 4 | 4 | 8 | 4 | 4 | 4 | 4 | 4 | 4 | 4 | 4 | 4 | 4 | 4 |
| **Total fat content** | 4 | 4 | 4 | 4 | 4 | 4 | 2 | 4 | 4 | 4 | 4 | 4 | 4 | 4 | 4 | 4 | 0 |
| **SFA** | 4 | 2 | 4 | 4 | 4 | 4 | 4 | 4 | 4 | 4 | 4 | 4 | 4 | 4 | 4 | 4 | 4 |
| **Palmitoleic acid** | 4 | 2 | 4 | 4 | 4 | 4 | 4 | 4 | 4 | 4 | 4 | 4 | 4 | 4 | 4 | 4 | 4 |
| **Palmitic acid** | 4 | 4 | 4 | 4 | 4 | 4 | 4 | 4 | 4 | 4 | 4 | 4 | 6 | 4 | 4 | 4 | 4 |
| **Butyrylcarnitine** | 4 | 4 | 4 | 4 | 4 | 4 | 4 | 4 | 4 | 4 | 4 | 4 | 9 | 4 | 4 | 4 | 4 |
| **Hexanoylcarnitine** | 4 | 4 | 4 | 4 | 4 | 4 | 4 | 4 | 4 | 4 | 4 | 4 | 6 | 4 | 4 | 4 | 4 |
| **DeoxyCer** | 4 | 4 | 4 | 4 | 4 | 4 | 4 | 4 | 4 | 4 | 4 | 4 | 6 | 4 | 4 | 4 | 4 |
| **DHC** | 4 | 4 | 4 | 4 | 4 | 4 | 4 | 4 | 4 | 4 | 4 | 4 | 9 | 4 | 4 | 4 | 4 |
| **Plasmalogenes** | 4 | 4 | 4 | 4 | 4 | 4 | 4 | 4 | 4 | 4 | 4 | 4 | 10 | 4 | 4 | 4 | 4 |
| **pPE** | 4 | 0 | 4 | 4 | 4 | 4 | 4 | 4 | 4 | 4 | 2 | 4 | 4 | 4 | 4 | 4 | 4 |
| **Total fucosylated HMOs** | 4 | 4 | 4 | 4 | 4 | 4 | 4 | 4 | 2 | 4 | 4 | 4 | 2 | 2 | 4 | 4 | 4 |
| **2`-FL** | 4 | 4 | 4 | 4 | 4 | 4 | 4 | 4 | 4 | 4 | 4 | 4 | 2 | 2 | 4 | 4 | 4 |
| **3-FL** | 4 | 4 | 2 | 4 | 4 | 4 | 4 | 4 | 4 | 4 | 4 | 4 | 4 | 4 | 2 | 2 | 4 |
| **5130c** | 4 | 4 | 4 | 4 | 4 | 4 | 4 | 4 | 4 | 4 | 4 | 4 | 1 | 4 | 4 | 4 | 4 |
| **Total non-fucosylated neutral HMOs** | 4 | 4 | 4 | 4 | 4 | 4 | 4 | 4 | 4 | 4 | 4 | 4 | 1 | 4 | 4 | 4 | 4 |
| **LNT** | 4 | 1 | 1 | 4 | 4 | 4 | 4 | 4 | 4 | 4 | 4 | 1 | 1 | 4 | 4 | 4 | 4 |
| **LNnT** | 4 | 4 | 4 | 4 | 4 | 4 | 4 | 4 | 4 | 4 | 4 | 4 | 1 | 4 | 4 | 4 | 4 |
| **LNH** | 4 | 4 | 4 | 4 | 4 | 4 | 4 | 4 | 4 | 4 | 4 | 4 | 1 | 4 | 4 | 4 | 4 |
| **pLNH** | 4 | 4 | 4 | 4 | 4 | 4 | 4 | 4 | 4 | 4 | 4 | 4 | 1 | 4 | 4 | 4 | 4 |
| **IFLNH I** | 4 | 4 | 4 | 4 | 4 | 4 | 4 | 4 | 4 | 4 | 4 | 4 | 1 | 4 | 4 | 4 | 4 |
| **5300 a** | 4 | 4 | 4 | 4 | 4 | 4 | 4 | 4 | 4 | 4 | 4 | 4 | 1 | 4 | 4 | 4 | 4 |
| **Total sialylated HMOs** | 4 | 4 | 4 | 4 | 4 | 4 | 4 | 4 | 4 | 4 | 4 | 4 | 2 | 4 | 4 | 4 | 4 |
| **3´-SL** | 4 | 4 | 4 | 4 | 4 | 4 | 4 | 4 | 4 | 4 | 2 | 2 | 2 | 4 | 4 | 4 | 4 |
| **6´-SL** | 4 | 4 | 1 | 4 | 4 | 4 | 4 | 4 | 4 | 4 | 4 | 4 | 4 | 4 | 4 | 4 | 4 |
| **5311a** | 4 | 4 | 4 | 2 | 4 | 4 | 4 | 4 | 4 | 4 | 4 | 4 | 4 | 4 | 4 | 4 | 4 |
| **LSTc** | 4 | 4 | 4 | 4 | 4 | 4 | 4 | 4 | 4 | 1 | 4 | 4 | 4 | 4 | 4 | 4 | 4 |
| **LSTb** | 4 | 4 | 4 | 4 | 4 | 4 | 4 | 4 | 4 | 4 | 4 | 4 | 2 | 4 | 4 | 4 | 4 |
| **FLSTc** | 4 | 4 | 4 | 2 | 4 | 4 | 4 | 4 | 4 | 4 | 4 | 4 | 4 | 4 | 4 | 4 | 4 |
| **Lactoferrin** | 4 | 4 | 4 | 1 | 4 | 4 | 4 | 4 | 4 | 4 | 4 | 4 | 4 | 4 | 4 | 4 | 4 |
| **Osteopontin** | 4 | 4 | 1 | 4 | 4 | 4 | 4 | 4 | 4 | 4 | 4 | 4 | 4 | 4 | 4 | 4 | 4 |
| **IgA** | 4 | 4 | 4 | 1 | 4 | 4 | 4 | 4 | 4 | 4 | 4 | 4 | 4 | 4 | 4 | 4 | 4 |
| **Lactalbumin** | 4 | 4 | 4 | 2 | 4 | 4 | 4 | 4 | 4 | 4 | 4 | 4 | 4 | 4 | 4 | 4 | 4 |
| **β-carotene** | 4 | 2 | 4 | 4 | 4 | 4 | 4 | 4 | 4 | 4 | 4 | 4 | 4 | 4 | 4 | 4 | 4 |
| **Lutein + Zeaxanthin** | 4 | 0 | 4 | 4 | 4 | 4 | 4 | 4 | 4 | 4 | 4 | 4 | 4 | 4 | 4 | 4 | 4 |
| **Lycopene** | 4 | 0 | 4 | 4 | 4 | 4 | 4 | 4 | 4 | 4 | 4 | 4 | 4 | 4 | 4 | 4 | 4 |
| **NAD+** | 4 | 4 | 4 | 4 | 4 | 4 | 4 | 4 | 4 | 4 | 4 | 4 | 2 | 4 | 4 | 4 | 4 |
| **Caffeine** | 4 | 4 | 4 | 4 | 4 | 4 | 4 | 4 | 4 | 4 | 4 | 4 | 1 | 4 | 4 | 4 | 4 |

0 = no association; 1 = negative association; 2 = positive association; 3 = double positive association; 4 = not available; 5 = no association girls; 6 = negative association girls; 7 = positive association girls; 8 = no association boys; 9 = negative association boys; 10 = positive association boys.
